# Supplementary material for: Ecological-level factors associated with tuberculosis incidence and mortality: A systematic review and meta-analysis
Source: PLOS Glob Public Health. 2024 Oct 15;4(10):e0003425. doi: 10.1371/journal.pgph.0003425 (PMC11478872; doi:10.1371/journal.pgph.0003425)
Supplement: S1 Table — (DOCX) [file pgph.0003425.s001.docx]

## S1 Table: Search strategies for ecological factors of TB incidence and mortality.

| Search | Query |
| --- | --- |
| **PubMed** | |
| #1 | tuberculosis OR TB OR mycobacterium tuberculosis OR MTB OR pulmonary tuberculosis OR PTB OR extrapulmonary tuberculosis OR EPTB |
| #2 | Incidence OR incidence rate OR cumulative incidence OR prevalence OR proportion OR mortality OR mortality rate |
| #3 | ecologic OR ecological OR ecologic level OR ecological factor OR ecologic factors OR environment OR environmental factors OR climate change OR climatic OR climatic factors OR meteorological factors OR air pollution OR air pollutants OR air quality related factors |
| #4 | #1 AND #2 AND #3 |
| Limited to | Humans |
| **SCOPUS** | |
| #1 | tuberculosis OR TB OR mycobacterium tuberculosis OR MTB OR pulmonary tuberculosis OR PTB OR extrapulmonary tuberculosis OR EPTB |
| #2 | Incidence OR incidence rate OR cumulative incidence OR prevalence OR proportion OR mortality OR mortality rate |
| #3 | ecologic OR ecological OR ecologic level OR ecological factor OR ecologic factors OR environment OR environmental factors OR climate change OR climatic OR climatic factors OR meteorological factors OR air pollution OR air pollutants OR air quality related factors |
| #4 | #1 AND #2 AND #3 |
| **Web of Science** | |
| #1 | tuberculosis OR TB OR mycobacterium tuberculosis OR MTB OR pulmonary tuberculosis OR PTB OR extrapulmonary tuberculosis OR EPTB |
| #2 | ecologic OR ecological OR ecologic level OR ecological factor OR ecologic factors OR environment OR environmental factors OR climate change OR climatic OR climatic factors OR meteorological factors OR air pollution OR air pollutants OR air quality related factors |
| #3 | Incidence OR incidence rate OR cumulative incidence OR prevalence OR proportion OR mortality OR mortality rate |
| #4 | #1 AND #2 AND #3 |
| Refined by | Environmental sciences ecology |
| **EMBASE** | |
| #1 | tuberculosis OR TB OR mycobacterium tuberculosis OR MTB OR pulmonary tuberculosis OR PTB OR extrapulmonary tuberculosis OR EPTB |
| #2 | Incidence OR incidence rate OR cumulative incidence OR prevalence OR proportion OR mortality OR mortality rate |
| #3 | ecologic OR ecological OR ecologic level OR ecological factor OR ecologic factors OR environment OR environmental factors OR climate change OR climatic OR climatic factors OR meteorological factors OR air pollution OR air pollutants OR air quality related factors |
| #4 | #1 #2 AND #3 |
