# Supplementary material for: Ecological-level factors associated with tuberculosis incidence and mortality: A systematic review and meta-analysis
Source: PLOS Glob Public Health. 2024 Oct 15;4(10):e0003425. doi: 10.1371/journal.pgph.0003425 (PMC11478872; doi:10.1371/journal.pgph.0003425)
Supplement: S2 Table — (DOCX) [file pgph.0003425.s002.docx]

**S2 Table:** Descriptive summary table for included studies.

| **First author (Year)** | **Country** | **Type of**  **geographic level** | **Data sources** | **Quality score** | **Quality judgement** |
| --- | --- | --- | --- | --- | --- |
| Alene (2019) | Ethiopia | District | Nationwide notified TB data, EDHS | 17 | High |
| Alene (2022) | Ethiopia | Cluster | TB prevalence survey, WorldClim website | 18 | High |
| Alene (2017) | Ethiopia | District | TB Control and Prevention Office, census | 18 | High |
| Alene (2017) | Ethiopia | District | Data from TB treatment centre and census | 18 | High |
| Alene (2021) | China | County | TB Control Institute, Worldclim database | 20 | High |
| Alves (2020) | Brazil | HDU | Mortality Information System | 17 | High |
| Amsalu (2019) | China | Province | CDC data and data from Bureau of Statistics | 17 | High |
| Arcoverde (2018) | Brazil | Census sectors | Mortality Information System and Census | 12 | Low |
| Bie (2021) | China | Province | TB surveillance, Meteorological, GDP data | 18 | High |
| Cao (2016) | China | Province | National Population Health Science and Meteorological Data | 17 | High |
| Carrasco-Escobar (2020) | Peru | District | TB data from health centres, Air quality data from the Socioeconomic database | 16 | High |
| Chen (2023) | China | District | TB surveillance data and population data | 17 | High |
| Couceiro (2011) | Portugal | Municipality | Survey (surveillance) and census | 14 | Low |
| Cui (2019) | China | Districts | TB-designated hospitals, Weather Bureau | 19 | High |
| da Roza (2012) | Brazil | Local areas | TB Notification system, Statistics Institute | 13 | Low |
| de Aberu (2016) | Brazil | Census tracts | National Brazilian database | 19 | High |
| Feske (2011) | US Texas | Census tracts | Surveillance data | 20 | High |
| Gelaw (2019) | Ethiopia | District | TB notification data and census data | 16 | High |
| Sousa (2022) | Brazil | Municipalities | Notifiable Diseases Information System | 20 | High |
| GUO (2017) | China | province | Centre for Disease Control and Prevention | 20 | High |
| Mohidem (2021) | Malaysia | Village | TB Information System database, Meteorological Department | 19 | High |
| He (2020) | China | Province | PTB surveillance system database | 19 | High |
| Im (2021) | South Korea | county | CDC | 20 | High |
| Li (2022) | China | Municipality | centre for Disease Control and Prevention | 21 | High |
| Li (2014) | China | Provinces | National TB Control Programme | 20 | High |
| Liu (2020) | China | Prefectures | CDC, Environmental Air Monitoring data | 19 | High |
| Munch (2003) | South Africa | subdistrict | Data from national TB program | 17 | High |
| Rao (2016) | China | County | TB incidence data and meteorological data | 19 | High |
| Rasam (2019) | Malaysia | Not reported | State Health Departments | 14 | Low |
| Sadeq (2018) | Morocco | provinces | TB surveillance data | 19 | High |
| Ghadimi (2020) | Iran | province | Health Center and meteorological database | 20 | High |
| Sohn (2019) | South Korea | Districts | Community Health Survey, NIER | 20 | High |
| Sohn (2019) | South Korea | Districts | Causes of Death Statistics, NIER | 20 | High |
| Sun (2015) | China | municipality | CDC and Meteorological Data | 19 | High |
| Wang (2019) | Mongolia | Counties | CDC | 19 | High |
| Zhang (2019) | China | Prefecture | CDC and Meteorological Data | 19 | High |
| Wei (2016) | China | District | TB surveillance system | 19 | High |
| Wubuli (2015) | China | District | CDC | 19 | High |
| Chaw (2022) | Brunei | NA | TB Coordinating Centre and weather station | 14 | Low |
| Chen (2016) | Taiwan | NA | Data from chest clinic record review | 18 | High |
| Huang (2020) | China | NA | TB Institute and Meteorological Data | 17 | High |
| Hwang (2014) | South Korea | NA | air monitoring stations and TB Institute | 14 | Low |
| Jassal (2013) | California | NA | Medical records and online Air Data | 18 | High |
| Kim (2020) | Korea | NA | TB Surveillance and air quality data | 17 | High |
| Kuddus (2019) | Bangladesh | NA | TB case notifications and Weather data | 19 | High |
| Lai (2016) | Taiwan | NA | Community-based screening service | 20 | High |
| Li (2023) | China | NA | Universal Health Examination Program data | 21 | High |
| Peng (2017) | China | NA | Data from TB registry and reporting system | 13 | Low |
| Nie (2022) | China | NA | PTB data and meteorological data | 14 | Low |
| Smith (2016) | California | NA | Air Monitoring Network Plan and TB data | 21 | High |
| Wang (2021) | China | NA | National Infectious Diseases Network | 19 | High |
| Zhu (2018) | China | NA | TB surveillance system, Environmental data | 18 | High |
|  |  |  |  | 21 | High |
