# Supplementary material for: Ecological-level factors associated with tuberculosis incidence and mortality: A systematic review and meta-analysis
Source: PLOS Glob Public Health. 2024 Oct 15;4(10):e0003425. doi: 10.1371/journal.pgph.0003425 (PMC11478872; doi:10.1371/journal.pgph.0003425)
Supplement: S3 Table — (DOCX) [file pgph.0003425.s003.docx]

**S3 Table:** a summary of the pooled effect estimates for factors associated with TB incidence examined in studies reporting odds ratios.

| **Factor** | **Number of studies** | **Odds Ratio (95%CI)** | **Heterogeneity (I^2^)** |
| --- | --- | --- | --- |
| Average temperature | 2 | 1.01(0.88,1.15) | 17.68% |
| Nitrogen dioxide | 3 | 1.10(0.99,1.21) | 82.23% |
| Carbon monoxide | 2 | **1.25(1.21,1.29)** | 00.00% |
| PM10 | 2 | **1.03(1.02,1.05)** | 0.00% |
| PM2.5 | 3 | 0.94(-0.49,2.38 | 91.63% |
