# Supplementary material for: Ecological-level factors associated with tuberculosis incidence and mortality: A systematic review and meta-analysis
Source: PLOS Glob Public Health. 2024 Oct 15;4(10):e0003425. doi: 10.1371/journal.pgph.0003425 (PMC11478872; doi:10.1371/journal.pgph.0003425)
Supplement: S4 Table — (DOCX) [file pgph.0003425.s004.docx]

**S4 Table:** Egger’s regression test results.

| **Factor** | **Egger’s regression test p-value** |
| --- | --- |
| Relative humidity | 0.025* |
| Average rainfall | 0.006* |
| Average wind speed | 0.416 |
| Sulfer dioxide | 0.937 |
| Fine particulate matter (PM2.5) | 0.070 |
| Coarse particulate matter (PM10) | NA |
| Carbon monoxide | 0.836 |
| Population density | NA |
| **Factor** | **Relative risk after trim and fill** |
| Relative humidity | RR=1.188, 95%CI:0.87,1.51 |
| Average rainfall | RR=1.28,95%CI:0.78,1.78 |

**Note:** *Trim and fill analysis was done; NA: not applicable
