# Supplementary material for: Ecological-level factors associated with tuberculosis incidence and mortality: A systematic review and meta-analysis
Source: PLOS Glob Public Health. 2024 Oct 15;4(10):e0003425. doi: 10.1371/journal.pgph.0003425 (PMC11478872; doi:10.1371/journal.pgph.0003425)
Supplement: S5 Table — (DOCX) [file pgph.0003425.s005.docx]

## **S5 Table:** Adapted quality assessment tool template and definitions.

| Evaluation criterion | Categories | Definition | Points (max=21) |
| --- | --- | --- | --- |
| STUDY DESIGN (max=12) | | | |
| Design | ecologic  multi-level design | If it is a multi-level design (e.g. ecologic + individual), the study is upgraded one point (e.g. ecologic + multi-level receives 2 points) | 1  2 |
| Sample size | < 80% units  ≥ 80% units | Number of ecologic units included in the analysis as a proportion of the total number of units, e.g., 50 districts of a total of 80 national districts would be 62.5%. | 0  1 |
| Unbiased inclusion of units | No  Yes | Were the units included representative of the group for which inferences are being drawn? For example, for national inferences, the inclusion of only developed regions or cities would be biased. | 0  1 |
| Level of data aggregation | Other than below  Regional, State  National | Population to which the units refer to. “Other” may be: city, race groups. | 1  2  3 |
| Level of inference | Individual or unclear  Ecologic | Use of the results of the analysis of the study’s sample data to draw inferences for individuals or groups (ecologic). | 0  1 |
| Pre-specification of ecologic units | No  Yes | Where the ecologic units selected to suit the hypothesis? (as opposed to selection motivated by convenience or necessity) | 0  1 |
| Outcomes of interest included | unclear  clear | A clear statement of relevant outcomes (i.e. type of TB, TB mortality,) | 1  2 |
| Source of data | Inadequate  Adequate | Validity of the sources of data to represent the level that it refers to (e.g., that data from one single hospital in one city would be an inadequate source of data to represent the national TB burden). | 0  1 |
| STATISTICAL METHODOLOGY (max=6) | | | |
| Analytic methodology | Bayesian Spatial poison regression, Bayesian spatial logistic regression, Bayesian spatiotemporal models, spatial panel models, spatial lag and error models, Eigenvector spatial regression model, OLS, GWR, Bayesian conditional autoregressive model, Bayesian negative binomial regression model, Regression Tree analysis | All statistical methods are acceptable as long as they are used appropriately. We assign a score based on the sophistication and flexibility of the method.  1 = local indicators of spatial association (LISA), OLS, Exposome-wide association study.  2 = all other models | 1  2 |
| Validity of regression | No  Yes | Did the adjustment have at least 10 units per covariate? | 0  1 |
| Use of covariates | Socioeconomic and demographic  Environmental or Climatic | The authors adjusted the analysis for desirable variables. Examples aggregated population level covariates: GDP, proportion of illiterate, unemployment rate, proportion of male. examples of Environmental or Climatic: average temperature average precipitation, average rain fall, particulate matter, sulphur dioxide. | 1  2 |
| Proper adjustment for covariates (yes) | No  Yes | Are the outcomes standardized or adjusted for certain factors before model adjustment? For standardized or adjusted outcomes, the standardized or adjusted factors should be included in the adjustment model. If standardized/adjusted outcomes are not used, this criterion is considered to have been met. | 0  1 |
| QUALITY OF REPORTING (max=3) | | | |
| Statement of study design (yes) | No  Yes | Did the authors present key elements of study design in the paper? | 0  1 |
| Justification of study design (yes) | No  Yes | Did the authors justify the ecologic analysis, the rationale and the specific objectives, including any prespecified hypotheses? | 0  1 |
| Discussion of cross-level bias and limitations (yes) | No  Yes | Did the authors caution readers about the limitations of the ecologic design, the ecologic fallacy, and the impossibility of extrapolating to a different level? | 0  1 |
