# Supplementary material for: Ecological-level factors associated with tuberculosis incidence and mortality: A systematic review and meta-analysis
Source: PLOS Glob Public Health. 2024 Oct 15;4(10):e0003425. doi: 10.1371/journal.pgph.0003425 (PMC11478872; doi:10.1371/journal.pgph.0003425)
Supplement: S6 Table — (DOCX) [file pgph.0003425.s006.docx]

**S6 Table: A numbered table of all studies identified in the literature search, with the full title of each article, including those that were excluded from the analyses.**

| ***NO*** | ***First author*** | ***Year of publication*** | ***Title*** | ***Name of Journal*** | ***Status*** | ***Reasons of exclusion*** |
| --- | --- | --- | --- | --- | --- | --- |
| *1* | *Bie* | *2021* | *Influential factors and spatial-temporal distribution of tuberculosis in mainland China* | *BMC Infectious Disease* | *Included* | *NA* |
| *2* | *Sohn* | *2019* | *Association of social deprivation and outdoor air pollution with pulmonary tuberculosis in spatiotemporal analysis.* | *IJEHR* | *Included* | *NA* |
| *3* | *Arcoverde* |  | *How do social-economic differences in urban areas affect tuberculosis mortality in a city in the tri-border region of Brazil, Paraguay and Argentina.* | *BMC Public Health* | *Included* | *NA* |
| *4* | *Alves,* | *2020* | *Bayesian spatio-temporal models for mapping TB mortality risk and its relationship with social inequities in a region from Brazilian Legal Amazon.* | *TRTMH* | *Included* | *NA* |
| *5* | *Ghadimi-Moghadam* |  | *Environmental and climatic factors influencing*  *the occurrence and distribution of tuberculosis in southwest iran: a gis-based study.* | *Acta Medica Mediterranea* | *Included* | *NA* |
| *6* | *Alene* | *2019* | *Spatial clustering of notified tuberculosis in Ethiopia: A nationwide study.* | *PLoS ONE* | *Included* | *NA* |
| *7* | *Alene* | *2022.* | *Spatial codistribution of HIV, tuberculosis and malaria in Ethiopia.* | *BMJ Glob Health* | *Included* | *NA* |
| *8* | *Alene* | *2017* | *Spatial patterns of multidrug resistant tuberculosis and relationships to socio-economic, demographic and household factors in northwest Ethiopia.* | *PLoS One* | *Included* | *NA* |
| *9* | *Alene* | *2017* | *Spatiotemporal transmission and socio-climatic factors related to paediatric tuberculosis in north-western Ethiopia.* | *Geospat Health* | *Included* | *NA* |
| *10* | *Alene* | *2021* | *Spatiotemporal Patterns of Tuberculosis in Hunan Province, China.* | *IJERPH* | *Included* | *NA* |
| *11* | *Amsalu* | *2019* | *Spatial-temporal analysis of tuberculosis in the geriatric population of China: An analysis based on the Bayesian conditional autoregressive model.* | *Archives of Gerontology & Geriatrics* | *Included* | *NA* |
| *12* | *Cao* | *2016* | *Spatial-Temporal Epidemiology of Tuberculosis in Mainland China: An Analysis Based on Bayesian Theory.* | *IJERPH* | *Included* | *NA* |
| *13* | *Carrasco-Escobar* | *2020* | *Spatio-temporal co-occurrence of hotspots of tuberculosis, poverty and air pollution in Lima, Peru.* | *Infectious Diseases of Poverty* | *Included* | *NA* |
| *14* | *Chen* | *2023* | *A Spatio-temporal Bayesian model to estimate risk and influencing factors related to tuberculosis in Chongqing, China, 2014-2020.* | *Archives of Public Health* | *Included* | *NA* |
| *15* | *Couceiro* | *2011* | *Santana, and C. Nunes, Pulmonary tuberculosis and risk factors in Portugal: A spatial analysis.* | *International Journal of Tuberculosis and Lung Disease* | *Included* | *NA* |
| *16* | *Cui* | *2019* | *Spatiotemporal patterns and ecological factors of tuberculosis notification: A spatial panel data analysis in Guangxi, China.* | *PLoS One* | *Included* | *NA* |
| *17* | *Da Roza* | *2012* | *Spatio-temporal patterns of tuberculosis incidence in Ribeirao Preto, State of Sao Paulo, southeast Brazil, and their relationship with social vulnerability: A Bayesian analysis.* | *Revista da Sociedade Brasileira de Medicina Tropical* | *Included* | *NA* |
| *18* | *De Abreu* | *2016* | *Spatial distribution of tuberculosis from 2002 to 2012 in a midsize city in Brazil.* | *BMC Public Health* | *Included* | *NA* |
| *19* | *Feske* | *2011* | *Including the third dimension: A spatial analysis of TB cases in Houston Harris County.* | *Tuberculosis* | *Included* | *NA* |
| *20* | *Gelaw* | *2019* | *Sociodemographic profiling of tuberculosis hotspots in Ethiopia, 2014-2017.* | *TRTMH* | *Included* | *NA* |
| *21* | *Guo* | *2017* | *Spatiotemporal analysis of tuberculosis incidence and its associated factors in mainland China.* | *Rev Bras Epidemiol.* | *Included* | *NA* |
| *22* | *He* | *2020* | *Spatial inequality, characteristics of internal migration, and pulmonary tuberculosis in China, 2011-2017: a spatial analysis.* | *Infectious Diseases of Poverty* | *Included* | *NA* |
| *23* | *Im* | *2021* | *Spatial pattern of tuberculosis (TB) and related socio-environmental factors in South Korea, 2008-2016.* | *PLOS ONE* | *Included* | *NA* |
| *24* | *Li* | *2022* | *Spatio-temporal distribution of tuberculosis and the effects of environmental factors in China.* | *Bmc Infectious Diseases* | *Included* | *NA* |
| *25* | *Li* | *2014* | *Exploration of ecological factors related to the spatial heterogeneity of tuberculosis prevalence in P. R. China.* | *Glob Health Action* | *Included* | *NA* |
| *26* | *Liu* | *2020* | *Associations of ambient air pollutants with regional pulmonary tuberculosis incidence in the central Chinese province of Hubei: a Bayesian spatial-temporal analysis.* | *Environmental Health* | *Included* | *NA* |
| *27* | *Mohidem* |  | *Association of sociodemographic and environmental factors with spatial distribution of tuberculosis cases in Gombak, Selangor, Malaysia. 2021.* | *Plos one* | *Included* | *NA* |
| *28* | *Munch* | *2003* | *Tuberculosis transmission patterns in a high-incidence area: a spatial analysis. International,* | *Journal of Tuberculosis and Lung Disease* | *Included* | *NA* |
| *29* | *Rao* | *2016* | *Spatial transmission and meteorological determinants of tuberculosis incidence in Qinghai Province, China: a spatial clustering panel analysis.* | *Infectious Disease Poverty* | *Included* | *NA* |
| *30* | *Rasam* | *2019* | *Spatial and Statistics for Profiling Risk Factors of Diseases: A Case Study of Tuberculosis in Malaysia.* | *Earth Environment* | *Included* | *NA* |
| *31* | *Sadeq* |  | *Spatiotemporal distribution and predictors of tuberculosis incidence in Morocco.* | *Infectious Disease Poverty* | *Included* | *NA* |
| *32* | *Sousa* | *2022* | *Spatiotemporal pattern of the incidence of tuberculosis and associated factors.* | *Revista Brasileira de Epidemiologia* | *Included* | *NA* |
| *33* | *Sun* | *2015* | *A spatial, social and environmental study of tuberculosis in China using statistical and GIS technology.* | *IJERPH* | *Included* | *NA* |
| *34* | *Wang* | *2019* | *Spatiotemporal epidemiology of, and factors associated with, the tuberculosis prevalence in northern China, 2010-2014.* | *BMC Infectious Disease* | *Included* | *NA* |
| *35* | *Wei* | *2016* | *Local spatial variations analysis of smear-positive tuberculosis in Xinjiang using geographically weighted regression model.* | *BMC Public Health* | *Included* | *NA* |
| *36* | *Wubuli* | *2015* | *Socio-demographic predictors and distribution of pulmonary tuberculosis (TB) in Xinjiang, China: a spatial analysis.* | *PloS one* | *Included* | *NA* |
| *37* | *Zhang* | *2019* | *, Y., et al., Spatial distribution of tuberculosis and its association with meteorological factors in mainland China.* | *Rev Saude Publica* | *Included* | *NA* |
| *38* | *Chaw* | *2022* | *Association between climate variables and pulmonary tuberculosis incidence in Brunei Darussalam.* | *Scientific Report* | *Included* | *NA* |
| *39* | *Chen* | *2016* | *Particulate matter is associated with sputum culture conversion in patients with culture-positive tuberculosis.* | *Therapeutics and Clinical Risk Management* | *Included* | *NA* |
| *40* | *Huang* | *2020* | *Association between short-term exposure to ambient air pollutants and the risk of tuberculosis outpatient visits: A time-series study in Hefei, China.* | *Environmental Research* | *Included* | *NA* |
| *41* | *Hwang* | *2014* | *Impact of outdoor air pollution on the incidence of tuberculosis in the Seoul metropolitan area, South Korea.,* | *Korean Journal of International Medicine* | *Included* | *NA* |
| *42* | *Jassal* | *2013* | *Correlation of ambient pollution levels and heavily trafficked roadway proximity on the prevalence of smear-positive tuberculosis.* | *Public health* | *Included* | *NA* |
| *43* | *Kim* | *2020* | *Effects of particulate air pollution on tuberculosis development in seven major cities of Korea from 2010 to 2016: methodological considerations involving long-term exposure and time lag.* | *Epidemiology and health* | *Included* | *NA* |
| *44* | *Kuddus* | *2019* | *Delay effect and burden of weather-related tuberculosis cases in Rajshahi province, Bangladesh, 2007–2012.* | *Scientific reports* | *Included* | *NA* |
| *45* | *Lai* | *2016* | *Ambient air pollution and risk of tuberculosis: a cohort study.* | *Occupational Environmental Medicine* | *Included* | *NA* |
| *46* | *Li* | *2023* | *Outdoor environmental exposome and the burden of tuberculosis: Findings from nearly two million adults in northwestern China.* | *Journal of Hazard Mater* | *Included* | *NA* |
| *47* | *Nie* | *2022* | *Effects and Interaction of Meteorological Factors on Pulmonary Tuberculosis in Urumqi, China, 2013-2019.* | *Frontiers in Public Health* | *Included* | *NA* |
| *48* | *Peng* | *2017* | *Long-term exposure to ambient air pollution and mortality in a Chinese tuberculosis cohort.* | *IJHR* | *Included* | *NA* |
| *49* | *Smith* | *2016* | *Air pollution and pulmonary tuberculosis: a nested case–control study among members of a northern California health plan.* | *Environmental health perspectives* | *Included* | *NA* |
| *50* | *Wang* | *2021* | *, W., et al., Epidemiological characteristics of tuberculosis and effects of meteorological factors and air pollutants on tuberculosis in Shijiazhuang, China: A distribution lag non-linear analysis.* | *Environmental research* | *Included* | *NA* |
| *51* | *Zhang* | *2022* | *An ecological study of tuberculosis incidence in China, from 2002 to 2018.* | *Frontiers in Public Health* | *Included* | *NA* |
| *52* | *Zhu* | *2018* | *Ambient air pollutants are associated with newly diagnosed tuberculosis: a time-series study in Chengdu, China.* | *Science of the Total Environment* | *Included* | *NA* |
| *53* | *Acosta* | *2014* | *The Porto Alegre paradox: social determinants and tuberculosis incidence* | *Revista Brasileira Epidemiology* | *Excluded* | *The outcome not clearly reported* |
| *54* | *Alene* | *2019* | *Mapping tuberculosis treatment outcomes in Ethiopia* | *BMC Infectious Diseases* | *Excluded* | *Not outcome of interest* |
| *55* | *Alves* | *2020* | *Risk areas for tuberculosis among children and their inequalities in a city in Southeast Brazil* | *BMC pediatrics* | *Excluded* | *Descriptive study* |
| *56* | *Heysell* | *2021* | *An Ecological Study of Tuberculosis Incidence in China, From 2002 to 2018* | *Pathogens* | *Excluded* | *Descriptive study* |
| *57* | *Alves* | *2019* | *Detection of risk clusters for deaths due to tuberculosis specifically in areas of southern Brazil where the disease was supposedly a non-problem* | *BMC Infectious Diseases* | *Excluded* | *Descriptive study* |
| *58* | *Andrade* | *2021* | *Spatial analysis of risk areas for the development of tuberculosis and treatment outcomes* | *PLoS One* | *Excluded* | *Descriptive study* |
| *59* | *André* | *2020* | *Tuberculosis associated with the living conditions in an endemic municipality in the North of Brazil* | *Rev.Latino Am. Enfermagem* | *Excluded* | *Descriptive study* |
| *60* | *Asri* | *2021* | *Spatial patterns of lower respiratory tract infections and their association with fine particulate matter* | *Scientific Reports* | *Excluded* | *Not outcome of interest* |
| *61* | *Bai* | *2019* | *A GIS-Based Artificial Neural Network Model for Spatial Distribution of Tuberculosis across the Continental United States* | *IJERPH* | *Excluded* | *Descriptive study* |
| *62* | *Bakker* | *2015* | *Studying the spatial distribution and temporal trends of tuberculosis notifications in India* | *European Journal of Epidemiology* | *Excluded* | *No full text is available* |
| *63* | *Bastida* | *2017* | *Spatial analysis of bovine tuberculosis in the State of Mexico, Mexico* | *Veterinaria Italiana* | *Excluded* | *Non-human study* |
| *64* | *Beiranv* | *2016* | *Correlation Assessment of Climate and Geographic Distribution of Tuberculosis Using Geographical Information System (GIS)* | *Iranian Journal of Public Health* | *Excluded* | *Descriptive study* |
| *65* | *Brown* | *1947* | *The influence of social factors on the incidence of extrapulmonary tuberculous infection; an investigation of the environment of tuberculous patients in Lanarkshire, Scotland* | *Journal of hygiene* | *Excluded* | *Not outcome of interest* |
| *66* | *Beiranv* | *2014* | *Assessment of tuberculosis distribution by geographical information system in Khuzestan province: A brief report. [Persian]* | *Tehran University Medical Journal* | *Excluded* | *Non-English article* |
| *67* | *Cavalin* | *2020* | *TB-HIV co-infection: spatial and temporal distribution in the largest Brazilian metropolis* | *-* | *Excluded* | *Descriptive study* |
| *68* | *Chen* | *2019* | *The characteristics of spatial-temporal distribution and cluster of tuberculosis in Yunnan Province, China, 2005-2018* | *BMC Public Health* | *Excluded* | *Descriptive study* |
| *69* | *Chen* | *2022* | *Modeling and Predicting Pulmonary Tuberculosis Incidence and Its Association with Air Pollution and Meteorological Factors Using an ARIMAX Model: An Ecological Study in Ningbo of China* | *International Journal of Environmental Research and Public Health* | *Excluded* | *Not outcome of interest* |
| *70* | *Chen* | *2021* | *The association between extreme temperature and pulmonary tuberculosis in Shandong Province, China, 2005–2016: a mixed method evaluation* | *BMC Infectious Diseases* | *Excluded* | *irrelevant study design* |
| *71* | *Corbett* | *2022* | *Migration and descent, adaptations to altitude and tuberculosis in Nepalis and Tibetans* | *Evolution, Medicine, and Public Health* | *Excluded* | *Not outcome of interest* |
| *72* | *China* | *2020* | *Spatial distribution of Mycobacterium Tuberculosis in metropolitan Harare, Zimbabwe* | *Plos One* | *Excluded* | *Descriptive study* |
| *73* | *Crisan* | *2015* | *Spatio-temporal analysis of tuberculous infection risk among clients of a homeless shelter during an outbreak* | *IJTD* | *Excluded* | *Not outcome of interest* |
| *74* | *Duan* | *2022* | *Epidemiological Characteristics and Spatial-Temporal Analysis of Tuberculosis at the County-Level in Shandong Province, China, 2016-2020* | *Tropical Medicine and Infectious Disease* | *Excluded* | *Descriptive study* |
| *75* | *Dhanaraj* | *2015* | *Prevalence and risk factors for adult pulmonary tuberculosis in a metropolitan city of South India* | *PLoS One* | *Excluded* | *Individual level factors* |
| *76* | *Dowdy* | *2017* | *Designing and Evaluating Interventions to Halt the Transmission of Tuberculosis* | *Journal of Infectious Diseases* | *Excluded* | *Not outcome of interest* |
| *77* | *Giacometti* | *2021* | *Temporal trend of tuberculosis incidence and its spatial distribution in Macapá - Amapá* | *IJERPH* | *Excluded* | *Descriptive study* |
| *78* | *Ghio* | *2014* | *Particle exposures and infections* | *Infection* | *Excluded* | *Not outcome of interest* |
| *79* | *He* | *2017* | *Relationship between climatic factors and air quality with tuberculosis in the Federal District, Brazil, 2003-2012* | *IJERPH* | *Excluded* | *Not outcome of interest* |
| *80* | *Hino* | *2011* | *[Spatial patterns of tuberculosis and its association with living conditions in the city of Ribeirão Preto in the state of São Paulo]* | *Cien Saude Colet* | *Excluded* | *Non-English article* |
| *81* | *Hu* | *2022* | *Unbalanced Risk of Pulmonary Tuberculosis in China at the Subnational Scale: Spatiotemporal Analysis* | *-* | *Excluded* | *No full text is available* |
| *82* | *Huang* | *2017* | *Spatial-temporal analysis of pulmonary tuberculosis in the northeast of the Yunnan province, People's Republic of China* | *Infectious Diseases of Poverty* | *Excluded* | *Descriptive study* |
| *83* | *Huang* | *2018* | *Space-time clustering and associated risk factors of pulmonary tuberculosis in Southwest China* | *Infectious Diseases of Poverty* | *Excluded* | *Duplicate* |
| *84* | *Jacob* | *2010* | *Accounting for autocorrelation in multi-drug resistant tuberculosis predictors using a set of parsimonious orthogonal eigenvectors aggregated in geographic space* | *Geospatial Health* | *Excluded* | *Methodological paper* |
| *85* | *Jiang* | *2016* | *Clustering of Pulmonary Tuberculosis in Hamadan Province, western Iran: A Population-Based Cross-Sectional Study (2005-2013)* | *IJERPH* | *Excluded* | *Descriptive study* |
| *86* | *Jo* | *2021* | *Sub-district level correlation between tuberculosis notifications and socio-demographic factors in Dhaka City corporation, Bangladesh* | *Epidemiology and Infection* | *Excluded* | *Descriptive study* |
| *87* | *Kapwata* | *2017* | *Spatial distribution of extensively drug-resistant tuberculosis (XDR TB) patients in KwaZulu-Natal, South Africa* | *Plos One* | *Excluded* | *Descriptive study* |
| *88* | *Kausarian* | *2016* | *Spatial and temporal analysis of tuberculosis in Zhejiang Province, China, 2009-2012* | *IJERPH* | *Excluded* | *Descriptive study* |
| *89* | *Khaliq* | *2022* | *Spatial distribution and computational modeling for mapping of tuberculosis in Pakistan* | *Journal of Public Health* | *Excluded* | *Descriptive study* |
| *90* | *Kalonji* | *2016* | *Prevalence of tuberculosis and associated risk factors in the Central Prison of Mbuji-Mayi, Democratic Republic of Congo* | *Tropical Medical Health* | *Excluded* | *Individual level factors* |
| *91* | *Ko* | *2022* | *Adverse Effects of Air Pollution on Pulmonary Diseases* | *Tuberculosis respiratory disease* | *Excluded* | *Review* |
| *92* | *Leining* | *2020* | *Geospatial and hot spot analysis of paediatric tuberculosis infection in Bohol, Philippines* | *Epidemiology and Infection* | *Excluded* | *outcome not clearly reported* |
| *93* | *Lima* | *2019* | *Spatial and temporal analysis of tuberculosis in an area of social inequality in Northeast Brazil* | *BMC Public Health* | *Excluded* | *Descriptive study* |
| *94* | *Lin* | *2022* | *Risk-prone territories for spreading tuberculosis, temporal trends, and their determinants in a high-burden city from São Paulo State, Brazil* | *Medicine (Baltimore)* | *Excluded* | *Not outcome of interest* |
| *95* | *Liu* | *2018* | *Spatial and temporal clustering analysis of tuberculosis in the mainland of China at the prefecture level, 2005-2015* | *Infectious Diseases of Poverty* | *Excluded* | *Descriptive study* |
| *96* | *Laumbach* | *2012* | *Respiratory health effects of air pollution: update on biomass smoke and traffic pollution* | *Journal of Allergy Clinical Immunology* | *Excluded* | *Not outcome of interest* |
| *97* | *Lien* | *2009* | *Prevalence and risk factors for tuberculosis infection among hospital workers in Hanoi, Viet Nam* | *PLoS One* | *Excluded* | *Not outcome of interest* |
| *98* | *Liu* | *2021* | *Effect of ambient air pollution on tuberculosis risks and mortality in Shandong, China: a multi-city modeling study of the short- and long-term effects of pollutants* | *Environmental Science and Pollution Research* | *Excluded* | *Not outcome of interest* |
| *99* | *Ma* | *2022* | *Spatial-temporal Analysis of Tuberculosis at the Community Level in the Baoshan District, Shanghai 2014-2019* | *Biomedical environmental science* | *Excluded* | *Letter to Editor* |
| *100* | *Mao* | *2019* | *Analysis of spatial-temporal distribution characteristics of smear-positive pulmonary tuberculosis in China, 2004-2015* | *International Journal of Infectious Diseases* | *Excluded* | *Descriptive study* |
| *101* | *Min* | *2023* | *Ambient PM(2.5) exposures could increase risk of tuberculosis recurrence* | *Environmental Health and Preventive Medicine* | *Excluded* | *Not outcome of interest* |
| *102* | *Muwonge* | *2010* | *Prevalence and associated risk factors of mycobacterial infections in slaughter pigs from Mubende district in Uganda* | *Trop Anim Health Prod* | *Excluded* | *Nonhuman study* |
| *103* | *Massene* | *2013* | *[Spatiotemporal distribution of tuberculosis cases in the city of Saint-Louis Senegal from 2008-2011]* | *Rev Epidemiol Sante Publique* | *Excluded* | *Non-English article* |
| *104* | *Pompilio* | *2020* | *Ambient air pollution and respiratory bacterial infections, a troubling association: epidemiology, underlying mechanisms, and future challenges* | *Critical reviews in microbiology* | *Excluded* | *Not outcome of interest* |
| *105* | *Rasam* | *2017* | *Mapping Risk Areas of Tuberculosis Using Knowledge-Driven GIS Model in Shah Alam, Malaysia* | *Journal of Social Science and Humanities* | *Excluded* | *Descriptive study* |
| *106* | *Rasam* | *2016* | *identifying high-risk populations of tuberculosis using environmental factors and gis based multi-criteria decision-making method* | *International Archives of the Photogrammetry* | *Excluded* | *Outcome not clearly reported* |
| *107* | *Rodrigues* | *2017* | *Distribution of pulmonary tuberculosis in Rio de Janeiro (Brazil): a spatial analysis* | *Ciência & Saúde Coletiva* | *Excluded* | *Descriptive study* |
| *108* | *Roza* | *2012* | *Spatio-temporal patterns of tuberculosis incidence in Ribeirao Preto, State of Sao Paulo, southeast Brazil, and their relationship with social vulnerability: a Bayesian analysis* | *Revista Da Sociedade Brasileira de Medicina Tropical* | *Excluded* | *Duplicate* |
| *109* | *Slama* | *2010* | *Indoor solid fuel combustion and tuberculosis: is there an association?* | *International journal of TB and lung disease* | *Excluded* | *Not outcome of interest* |
| *110* | *Tabatabaee* | *2015* | *Spatio-Temporal Pattern of Tuberculosis in the Regions Supervised by Shiraz University of Medical Sciences 2006-2012* | *Iran Journal of Public Health* | *Excluded* | *Duplicate* |
| *111* | *Tabatabaee* | *2015* | *Determining spatial pattern of Tuberculosis by geographical information system in Guilan Province, 2005-2011. [Persian]* | *Journal of Mazandaran University of Medical Sciences* | *Excluded* | *No full text available* |
| *112* | *Tang* | *2023* | *Machine Learning Prediction Model of Tuberculosis Incidence Based on Meteorological Factors and Air Pollutants* | *Environmental Science and Pollution Research* | *Excluded* | *Not outcome of interest* |
| *113* | *Tian* | *2019* | *Spatial patterns and effects of air pollution and meteorological factors on hospitalization for chronic lung diseases in Beijing, China* | *Science China. Life sciences* | *Excluded* | *Not outcome of interest* |
| *114* | *Traoré* | *2022* | *Effectiveness of the Novel Anti-TB Bedaquiline against Drug-Resistant TB in Africa: A Systematic Review of the Literature* | *pathogens* | *Excluded* | *Review* |
| *115* | *Ullah* | *2020* | *Space-Time Clustering Characteristics of Tuberculosis in Khyber Pakhtunkhwa Province, Pakistan, 2015-2019* | *BMC Infectious disease* | *Excluded* | *Descriptive study* |
| *116* | *Wang* | *2012* | *The spatial epidemiology of tuberculosis in Linyi City, China, 2005-2010* | *BMC Public Health* | *Excluded* | *Descriptive study* |
| *117* | *Winkler* | *2015* | *Environmental risk factors associated with bovine tuberculosis among cattle in high-risk areas* | *Biology letters* | *Excluded* | *Nonhuman study* |
| *118* | *Yacoub* | *2011* | *Disease appearance and evolution against a background of climate change and reduced resources* | *Philos Transacions A Mathical Physical Engineering Sciences* | *Excluded* | *Not outcome of interest* |
| *119* | *Yang* | *2019* | *Spatiotemporal Distribution of Tuberculosis during Urbanization in the New Urban Area of Nanchang City, China, 2010-2018* | *International Journal of Infectious Diseases* | *Excluded* | *Descriptive study* |
| *120* | *Yao* | *2019* | *Ambient air pollution exposures and risk of drug-resistant tuberculosis* | *Environment international* | *Excluded* | *Not outcome of interest* |
| *121* | *Yasri,* | *2021* | *Tuberculosis incidence in area with sulfur dioxide pollution: an observation* | *Medical Gas Research* | *Excluded* | *Descriptive study* |
| *122* | *Yi* | *2021* | *Impact of environmental factors on pulmonary tuberculosis in multi-levels industrial upgrading area of China* | *Environmental research* | *Excluded* | *Not outcome of interest* |
| *123* | *Yu* | *2020* | *Spatial-temporal analysis of tuberculosis in Chongqing, China 2011-2018* | *BMC Infectious disease* | *Excluded* | *Descriptive study* |
| *124* | *Yun* | *2022* | *Time trend prediction and spatial-temporal analysis of multidrug-resistant tuberculosis in Guizhou Province, China, during 2014-2020* | *BMC Infectious disease* | *Excluded* | *Descriptive study* |
| *125* | *Zeng* | *2020* | *The Spatiotemporal Dynamic Distributions of New Tuberculosis in Hangzhou, China* | *Biomedical environmental science* | *Excluded* | *Letter to Editor* |
| *126* | *Zhang* | *2021* | *Space-time clustering and temporal trends of hospitalizations due to pulmonary tuberculosis: a potential strategy for assessing health care policies* | *IJERPH* | *Excluded* | *Descriptive study* |
| *127* | *Zhang* | *2015* | *Effect of meteorological factors on incidence of tuberculosis: A 15-year retrospective study based on Chinese medicine theory of five circuits and six qi* | *Environmental health* | *Excluded* | *Not outcome of interest* |
| *128* | *Zhao* | *2013* | *Space-Time Clustering Characteristics of Tuberculosis in China, 2005-2011* | *Plos One* | *Excluded* | *Descriptive study* |

**Note:** IJERPH: International Journal of Environmental Research and Public Health; IJEHR: International Journal of Environmental Health Research; IJTD: International Journal of Tuberculosis and Lung Disease; TRSTMH: Transactions of the Royal Society of Tropical Medicine and Hygiene;IJHR:INTERNATIONAL Journal of Health Research
