# Supplementary material for: Ecological-level factors associated with tuberculosis incidence and mortality: A systematic review and meta-analysis
Source: PLOS Glob Public Health. 2024 Oct 15;4(10):e0003425. doi: 10.1371/journal.pgph.0003425 (PMC11478872; doi:10.1371/journal.pgph.0003425)
Supplement: S2 Fig — (DOCX) [file pgph.0003425.s008.docx]

**S2 Fig:** Forest plot of pooled effect (odds ratio) of particulate matter (PM10) on TB incidence.
