# Supplementary material for: Ecological-level factors associated with tuberculosis incidence and mortality: A systematic review and meta-analysis
Source: PLOS Glob Public Health. 2024 Oct 15;4(10):e0003425. doi: 10.1371/journal.pgph.0003425 (PMC11478872; doi:10.1371/journal.pgph.0003425)
Supplement: Attachment — Submitted filename: Peer review PLOS.docx [file pgph.0003425.s012.docx]

**Review comments to the author**

This is a generally well-written article that has been clearly justified. Please see suggestions below to improve clarity and reporting.

Introduction:

1. I find the use of the term ecological factors slightly confusing as it is often associated with environmental factors. I think it would be useful for authors to clarify what they define as ‘ecological factors’ or consider using a more encompassing term such as ‘population-level factors’.

Methods:

1. Author stated in protocol that Newcastle Ottawa would be used to assess bias. Please justify the reason for using a different risk of bias tool and transparently report differences between protocol and review.
2. Unclear why ref number 37 is included in the risk of bias section.
3. “Narrative synthesis was used to qualitatively summarize the effect of ecological factors on the primary outcome of interest including TB incidence and mortality” This isn’t very clear – please could the authors provide more detail regarding the methods to summarise this data, see SWiM guidance <https://doi.org/10.1136/bmj.l6890>

Results:

1. Suggest streamlining table 1 as it is too large at present. I would include column 1, 3, 6 and 8 here. Risk of bias/quality results should be presented in a separate table with the narrative explanation of judgements. Other data could be reported in an Appendix.
2. The authors need to include denominators in their results – currently included sentences such as “TB incidence was consistently positively associated with mean temperature (n=10)”, but it is unclear whether only 10 studies measured this and all reported this association (in which case report as 10/10 studies) or whether 20 studies reported this outcome but the positive association was only seen in 10 studies (then report as 10/20 studies). This needs to be done throughout the results.
3. The authors have stated sentences such as “TB incidence was consistently positively associated with mean temperature (n=10), average humidity (n=9), precipitation (n=4), rainfall (n=4) and elevation (n=1).”. Do the mean that higher mean temperatures or lower mean temperatures are positively associated with TB incidence? Higher or lower precipitation? Etc. This needs to be clarified throughout the results.
4. For studies that report a conflicting result, such as the study in Mongolia that reported a negative association between TB and population density, are authors able to offer any explanation for this result?
5. Add denominators to table 2 and 3 to show total number of studies reporting each outcome.
6. I wonder if there is a more visual way to present the data in table 2 and 3, maybe a stacked bar chart?

Meta-analysis results:

1. Please explain definition of “methodologically suitable for inclusion in meta-analysis”.
2. Again, it is not clear whether *increased* or *decreased* humidity, rainfall, population density, etc are associated with increased risk of outcomes. Please clarify as this is important for the reader to understand and interpret the results.
3. Please narratively acknowledge the high I^2^ value associated with some results and discuss the implications of this.
4. In Table 4 please provide footnote explaining that results in bold represent significant results.

Quality assessment:

1. Please could authors provide a separate table here with results of risk of bias assessment and provide specific examples of issues with studies that resulted in low risk of bias scores. This allows the reader to understand common issues with these studies and aides’ interpretation of findings.

Discussion:

1. Suggest to add sub-headings for summary of evidence and limitations.

References:

1. Suggest separating included studies from other references if possible.

Supplementary files

1. Is it possible to add participant numbers, number of events, and risk of bias results to forest plots? See recent Cochrane reviews as an example.
2. Not recommended to conduct a funnel plot with fewer than 10 studies. Please consider whether these are reliable or useful.
3. Excluded studies – please be consistent with reason for exclusion such as using ‘irrelevant study design’ rather than ‘meta-analysis’
