# Supplementary material for: Ecological-level factors associated with tuberculosis incidence and mortality: A systematic review and meta-analysis
Source: PLOS Glob Public Health. 2024 Oct 15;4(10):e0003425. doi: 10.1371/journal.pgph.0003425 (PMC11478872; doi:10.1371/journal.pgph.0003425)
Supplement: Attachment — Submitted filename: Revised response to reviewers.docx [file pgph.0003425.s013.docx]

**Date 07/06/2024.**

**Manuscript reference: PGPH-D-24-00826**

**Title: Ecological-level factors associated with tuberculosis incidence and mortality: A systematic review and meta-analysis.**

***PLOS Global Public Health***

Dear all,

We would like to thank you for the constructive and helpful comments on this manuscript. Our point-by-point responses to each of the reviewer and editor comments are described in detail on the following pages. Further, the details of the changes are shown by tracked changes in the revised attached documents.

**Version I: Editor comments**

**Comment 1:** Please amend your detailed Financial Disclosure statement. This is published with the article. It must therefore be completed in full sentences and contain the exact wording you wish to be published. a. State the initials, alongside each funding source, of each author to receive each grant. b. State what role the funders took in the study. If the funders had no role in your study, please state: “The funders had no role in study design, data collection and analysis, decision to publish, or preparation of the manuscript.” c. If any authors received a salary from any of your funders, please state which authors and which funders. If you did not receive any funding for this study, please simply state: “The authors received no specific funding for this work.”

**Authors’ response:** We have revised the financial disclosure as per the above suggestion and included it in the revised manuscript. **Please see lines 429-432.**

**Comment 1:** Please provide separate figure files in .tif or .eps format only and remove any figures embedded in your manuscript file. Please also ensure all files are under our size limit of 10MB.

**Authors’ response:** Separate figure files are provided with tiff file format and recommended file size.

**Comment 1:** We have noticed that you have uploaded Supporting Information files, but you have not included a list of legends. Please add a full list of legends for your Supporting Information files after the references list.

**Authors’ response:** we have now included a list of legends for the supportive files next to reference in the manuscript.

**Comment 1:** In the online submission form, you indicated that "Data will be available upon request from the corresponding author". All PLOS journals now require all data underlying the findings described in their manuscript to be freely available to other researchers, either In a public repository, Within the manuscript itself, or uploaded as supplementary information.

**Authors’ response:** The data are now available within the manuscript.

**Version II: Reviewers’ comments**

**Reviewers’ Responses to general questions**

**Q1:** Does this manuscript meet PLOS Global Public Health’s [publication criteria](https://journals.plos.org/globalpublichealth/s/criteria-for-publication)? Is the manuscript technically sound, and do the data support the conclusions? The manuscript must describe methodologically and ethically rigorous research with conclusions that are appropriately drawn based on the data presented.

Reviewer #1: Yes

Reviewer #2: Yes

**Authors’ response:** Thank you.

**Q2:** Has the statistical analysis been performed appropriately and rigorously?

Reviewer #1: I don't know

Reviewer #2: Yes

**Authors’ response:** Thank you.

**Q3:** Have the authors made all data underlying the findings in their manuscript fully available (please refer to the Data Availability Statement at the start of the manuscript PDF file)?
The [PLOS Data policy](https://journals.plos.org/globalpublichealth/s/data-availability) requires authors to make all data underlying the findings described in their manuscript fully available without restriction, with rare exception. The data should be provided as part of the manuscript or its supporting information or deposited to a public repository. For example, in addition to summary statistics, the data points behind means, medians and variance measures should be available. If there are restrictions on publicly sharing data—e.g. participant privacy or use of data from a third party—those must be specified.

Reviewer #1: No

Reviewer #2: Yes

**Authors’ response:** The data are now fully available within the manuscript.

**Q4:** Is the manuscript presented in an intelligible fashion and written in standard English?
PLOS Global Public Health does not copyedit accepted manuscripts, so the language in submitted articles must be clear, correct, and unambiguous. Any typographical or grammatical errors should be corrected at revision, so please note any specific errors here.

Reviewer #1: Yes

Reviewer #2: Yes

**Authors’ response:** Thank you.

**Reviewer #1**

**Comment 1:** This is a generally well-written article that has been clearly justified. Please see suggestions below to improve clarity and reporting.

**Authors’ response:** Thank you for your comments and suggestions.

**Comment 2:** I find the use of the term ecological factors slightly confusing as it is often associated with environmental factors. I think it would be useful for authors to clarify what they define as ‘ecological factors’ or consider using a more encompassing term such as ‘population-level factors.’

**Authors’ response:** The term “ecological factor” is well understood in epidemiology to mean a factor measured at an aggregate level. In our manuscript, we have comprehensively considered the expansive realm of ecological factors, recognizing their diverse manifestations and impacts. We have provided some more information to define ecological factors as follows: “…….to identify studies that specifically investigated the association between ecological factors namely, climatic variables, environmental factors, and indicators measured at an aggregate population level…….” **lines 98-104 of the methods section**.

Thus, we have included ecological factors pertaining to abiotic elements, encapsulating climatic and environmental variables (1, 2) and other groups of ecological factors that encompass aggregated population-level socioeconomic and demographic characteristics (3, 4). These aggregated population-level factors offer a panoramic view of societal dynamics, reflecting broader patterns of inequality, accessibility, and resilience which couldn’t be addressed by individual level studies. Within this framework, socioeconomic indicators such as income, education, and employment status and demographic variables including population density, urbanization, and migration patterns and many others, collectively shape the ecological milieu in which diseases like TB proliferate.

**Comment 2:** Author stated in protocol that Newcastle Ottawa would be used to assess bias. Please justify the reason for using a different risk of bias tool and transparently report differences between protocol and review.

**Authors’ response:** The Newcastle-Ottawa Scale (NOS) is primarily designed for assessing the quality of non-randomized observational studies, particularly cohort and case-control studies, in systematic reviews and meta-analyses. However, ecological studies often have unique characteristics and methodological challenges that differ from individual-level observational studies. They typically analyze aggregated data at the population level rather than individual-level data. As a result, the criteria used in NOS, which are focused on individual-level study designs, don’t fully capture the specific biases and limitations inherent in ecological studies.

Instead, for assessing bias in ecological studies, we look for other methodologically appropriate tools or criteria that consider factors such as the ecological fallacy, potential confounding at the ecological level, validity of exposure and outcome measurements at the population level. Therefore, we used an adapted quality assessment checklist proposed by Dufault et al. (5) which evaluates aspects related to study design, statistical methodology and reporting quality of ecologic studies for better evaluation of studies included. This tool was also used in previous systematic review study involving ecological studies (6).

**Comment 2:** Unclear why ref number 37 is included in the risk of bias section.

**Authors’ response:** The reference has been removed to avoid confusion, and the paragraph is restructured for clarity. **Please see lines 147-152.**

**Comment 3:** “Narrative synthesis was used to qualitatively summarize the effect of ecological factors on the primary outcome of interest including TB incidence and mortality” This isn’t very clear – please could the authors provide more detail regarding the methods to summarise this data, see SWiM guidance <https://doi.org/10.1136/bmj.l6890>

**Authors’ response:** Further explanation on the type of narrative synthesis employed has now been provided, as follows: **“……** In this study, when a meta-analysis of effect estimates was not deemed appropriate due to incompatible effect sizes reported and different variable definitions used across studies, a narrative synthesis method called Synthesis Without Meta-analysis (SWiM), as outlined in the PRISMA extension guideline (36), was utilized. Firstly, studies were categorized based on the type of ecological factors they examined, such as climatic, air quality-related, and socioeconomic factors. Since pooling effect sizes via meta-analysis wasn't feasible due to the inconsistencies in reported effect sizes and variations in variable definitions across studies, we employed a narrative synthesis method called vote counting based on the direction of effect (36). This approach involved tallying the direction of association reported in each study for a particular ecological factor. For each ecological factor, the direction of association (positive, negative, or neutral) was noted based on the effect sizes reported in the individual studies. Summary tables and graphs were then created to present the overall direction of association for each factor across the included studies. Additionally, detailed narration was provided in the text, describing the number of studies that reported a given direction of association among all the studies that have investigated a particular factor.” **please refer to lines 154-174.**

**Comment 4:** Suggest streamlining table 1 as it is too large at present. I would include column 1, 3, 6 and 8 here. Risk of bias/quality results should be presented in a separate table with the narrative explanation of judgements. Other data could be reported in an Appendix.

**Authors’ response:** We have now split table 1 to aid the understanding of summarized findings. Keeping key findings in the main text, we pushed all other information to the Appendix as per the reviewer’s suggestion**. Please kindly refer to the table on pages 8-11.**

**Comment 5:** The authors need to include denominators in their results – currently included sentences such as “TB incidence was consistently positively associated with mean temperature (n=10)”, but it is unclear whether only 10 studies measured this and all reported this association (in which case report as 10/10 studies) or whether 20 studies reported this outcome but the positive association was only seen in 10 studies (then report as 10/20 studies). This needs to be done throughout the results.

**Authors’ response:** We have now included the denominator for each factor throughout the manuscript including tables and figures.

**Comment 6:** The authors have stated sentences such as “TB incidence was consistently positively associated with mean temperature (n=10), average humidity (n=9), precipitation (n=4), rainfall (n=4) and elevation (n=1).”. Is the meaning that higher mean temperatures or lower mean temperatures are positively associated with TB incidence? Higher or lower precipitation? Etc. This needs to be clarified throughout the results.

**Authors’ response:** The directions of associations are now clearly stated throughout the manuscript.

**Comment 7:** For studies that report a conflicting result, such as the study in Mongolia that reported a negative association between TB and population density, are authors able to offer any explanation for this result?

**Authors’ response:** A proposed explanation for the conflicting finding in Mongolia is now included.

**Comment 8:** Add denominators to table 2 and 3 to show total number of studies reporting each outcome.

**Authors’ response:** We have now added the total number of studies reporting each outcome**.**

**Comment 9:** I wonder if there is a more visual way to present the data in table 2 and 3, maybe a stacked bar chart?

**Authors’ response**: We have now presented variables associated with TB incidence and mortality including climatic and air quality related factors using a stacked bar graph as suggested by the reviewers. However, other variables were presented in a table format as they were too many to present in a figure format.

**Comment 10:** Please explain definition of “methodologically suitable for inclusion in meta-analysis”.

**Authors’ response:** we have now revised this sentence as follows: **“**A total of twenty-six studies (8-32) that examined the association between ecological factors and TB incidence and reported compatible effect sizes, were considered methodologically suitable for inclusion in the meta-analysis.” **Please refer to lines 198-200.**

**Comment 11:** Again, it is not clear whether increased or decreased humidity, rainfall, population density, etc are associated with increased risk of outcomes. Please clarify as this is important for the reader to understand and interpret the results.

**Authors’ response:** As above, directions of associations are now clear.

**Comment 12:** Please narratively acknowledge the high I2 value associated with some results and discuss the implications of this.

**Authors’ response:** We have now discussed the implications of high heterogeneity. **Please see lines 386-390 of the limitations section.**

**Comment 13:** In Table 4 please provide a footnote explaining that results in bold represent significant results.

**Authors’ response:** We have now included this in the footnote.

**Comment 14:** Please could authors provide a separate table here with results of risk of bias assessment and provide specific examples of issues with studies that resulted in low risk of bias scores. This allows the reader to understand common issues with these studies and aides’ interpretation of findings.

**Authors’ response:** We have now narratively described the common bias issues that appeared mostly in low quality articles. As suggested by the reviewer, we have also provided a separate table containing the quality score and other relevant information and presented it in the appendix. **please refer to lines 289-296.**

**Comment 15:** Suggest adding sub-headings for summary of evidence and limitations.

**Authors’ response:** we have now provided separate headings for limitations.

**Comment 16:** Suggest separating included studies from other references if possible.
Supplementary files.

**Authors’ response:** we have now separated included studies from other references.

**Comment 17:** Is it possible to add participant numbers, number of events, and risk of bias results to forest plots? See recent Cochrane reviews as an example.

**Authors’ response:** since we couldn’t locate a, b, c, d, for each ecological study due to a lack of data, it was not possible to produce forest plots containing participant numbers, number of events. This is more appropriate for meta-analysis summarizing individual level data. We used adjusted odds ratios and relative risks reported from fitted model outputs reported in primary studies to produce the forest plots.

**Comment 18:** Not recommended to conduct a funnel plot with fewer than 10 studies. Please consider whether these are reliable or useful.

**Authors’ response:** As suggested by the reviewer the funnel plots are now removed in the revised manuscript.

**Comment 19:** Excluded studies – please be consistent with reason for exclusion such as using ‘irrelevant study design’ rather than ‘meta-analyses’.

**Authors’ response:** We have now replaced meta-analysis with ‘irrelevant study design’.

**Comment 20:** Please make all data publicly available either in supporting information or via an online repository.

**Authors’ response:** The data are now publicly available in the manuscript.

**Reviewer #2**

**Comment 1:** The authors have produced a manuscript describing the ecological factors associated with TB incidence and mortality through the conduct of a systematic review and meta-analysis. The article is timely and attempts to narrow the gap in knowledge of factors associated with TB epidemiology. The authors should try to address the following:

**Authors’ response:** Thank you for your constructive comments. All the comments are addressed below.

**Comment 2:** The introduction fails to establish a strong rationale for the conduct of the study. Despite some inconsistencies in the literature about specific ecological factors associated with TB incidence and mortality, some consensus exists amongst TB researchers about these factors. There is thus a need to clearly outline why a comprehensive review on these factors is necessary to end the TB epidemic in line with End TB Strategy goals.

**Authors’ response:** We have now included an additional paragraph stating the implication of synthesizing the evidence on the impact of ecological factors on TB incidence and mortality in the context of End TB Strategy objectives. **Please kindly refer to lines 89-95 of the introduction.**

**Comment 3:** The methods do not clearly define the "ecological factors" of interest that determined the papers that were to be included in this review. A statement about the population of interest (adult versus paediatric populations) would also be useful in fulfilling the PICOS requirements. A look at Table 2, which summarizes the ecological factors extracted from the included studies alludes to socioeconomic factors such as poverty indicators, population density and HIV testing practices being included as ecological factors. Using this table as the general definition of ecological factors, a quick search on Google Scholar brings up papers by Qianyun Zhang et al. (study from China published in Frontiers in Public Health in 2021), and Soares P et al. (published in Public Health in 2022) among others which are not included in the manuscript, suggesting that the search strategy was inadequate, which may be a result of poorly defining the Population of interest (P), Intervention/Exposures (I) (i.e. the ecological factors of interest), Comparators (C), and the Outcomes (O) (i.e. the measures of disease occurrence of interest).
**Authors’ response:** No restriction was applied on the type of population included, as was already noted in the search strategy. This review includes adult and paedatric populations. We have now clarified this in the manuscript **lines 106-107 of the methods section**. A detailed explanation on the ecological factors included in this study was provided in our response to **comment 2** of reviewer 1 (we refer to the above explanation). Briefly, we considered common ecological factors such as climatic, environmental and population level data aggregated at a geographical scale and the search strategy was designed accordingly. Regarding “Soares P et al’s paper which was entitled “Ecological factors associated with areas of high tuberculosis diagnosis delay”, although it investigates ecological factors, the outcome of interest is “TB diagnosis delay.” For primary studies to be included in our study, the outcome of interest should be either “TB incidence” or “TB mortality.” So the above paper was excluded during title or abstract screening during our review process. However, we have now included “Qianyun Zhang et al, 2022”, which might have been missed because Google Scholar was not among the databases included in our initial search. Thank you for picking it up.

**Comment 4:** The authors produced forest plots for the meta-analysis outputs of individual ecological factors. It is understood that the journal has guidelines limiting the number of figures that may be included in the main manuscript. However, the forest plots are direct analytical outputs of the main objective of the paper, and as such should feature in the main paper, and not the supplementary materials. The authors could group the figures using panels to create one illustration with a good figure legend to facilitate the inclusion of these in the main manuscript.
**Authors’ response:** We have now assembled all forest plots within a panel figure and included them in the main manuscript.

**Comment 5:** Under the data extraction section on page 5, line 19: "analytyc" should be corrected to "analytic"

**Authors’ response:** Thank you. We have made the correction.

**Comment 6:** In outlining potential limitations of the study, the ecological fallacy was rightly mentioned. However, the authors fail to suggest what effects the presence of the ecological fallacy would potentially have on the results of the study, merely stating it could result in "misleading conclusions".

**Authors’ response:** we have now described how the ecological fallacy might affect the current results. **Please see line 395-400 of the limitations section.**

**Comment 7:** Figure S22: Quality of 52 included studies: provide units/axis title for the y (number) axis at the bottom.

**Authors’ response:** We have now corrected this in the revised manusicpt.

**References**

1.	Coker R, McKee M, Atun R, Dimitrova B, Dodonova E, Kuznetsov S, et al. Risk factors for pulmonary tuberculosis in Russia: case-control study. Bmj. 2006;332(7533):85-7.

2.	Ment D, Shikano I, Glazer I. Abiotic factors. Ecology of invertebrate diseases. 2017:143-86.

3.	Oxlade O, Murray M. Tuberculosis and poverty: why are the poor at greater risk in India? PloS one. 2012;7(11):e47533.

4.	Kandula S, Shaman J. Investigating associations between COVID-19 mortality and population-level health and socioeconomic indicators in the United States: A modeling study. PLoS Medicine. 2021;18(7):e1003693.

5.	Dufault B, Klar N. The quality of modern cross-sectional ecologic studies: a bibliometric review. American journal of epidemiology. 2011;174(10):1101-7.

6.	Betran AP, Torloni MR, Zhang J, Ye J, Mikolajczyk R, Deneux-Tharaux C, et al. What is the optimal rate of caesarean section at population level? A systematic review of ecologic studies. Reproductive health. 2015;12:1-10.

7.	Campbell M, McKenzie JE, Sowden A, Katikireddi SV, Brennan SE, Ellis S, et al. Synthesis without meta-analysis (SWiM) in systematic reviews: reporting guideline. bmj. 2020;368.

33.	Sterne JA, Egger M, Smith GD. Investigating and dealing with publication and other biases in meta-analysis. Bmj. 2001;323(7304):101-5.

34.	Egger M, Smith GD, Schneider M, Minder C. Bias in meta-analysis detected by a simple, graphical test. Bmj. 1997;315(7109):629-34.
