# Supplementary material for: Ecological-level factors associated with tuberculosis incidence and mortality: A systematic review and meta-analysis
Source: PLOS Glob Public Health. 2024 Oct 15;4(10):e0003425. doi: 10.1371/journal.pgph.0003425 (PMC11478872; doi:10.1371/journal.pgph.0003425)
Supplement: Attachment — Submitted filename: Repsonse to reviewers.docx [file pgph.0003425.s014.docx]

**Date 10/08/2024**

**Manuscript reference: PGPH-D-24-00826R1**

**Title: Ecological-level factors associated with tuberculosis incidence and mortality: A systematic review and meta-analysis.**

***PLOS Global Public Health***

Dear Editor,

We would like to thank you for the constructive and helpful comments on this manuscript. Our point-by-point responses to each of the editor's comments are described in detail on the following pages. Further, the details of the changes are shown by tracked changes in the revised attached documents.

**Comment 1:** Figure 1 is a useful schematic; however, for readers to be able to follow with the text, it would be good to use the same headings for subsection titles under “Methods”. E.g. the subsection “Data source and search strategy” up to line 120 of p.9 could be combined under a subsection titled “Identification”, lines 121 – 132 could be under a subsection titled “Screening” etc.

**Authors’ response:** We have now structured the method section as “Identification”, “Study Screening and Inclusion”, and “Data Extraction” as suggested. **Please see lines 100,120 and 135** respectively.

**Comment 2:** Figure 1: How do the items (“Full-text articles excluded”, “Descriptive studies” etc.) relate to the items in the quality assessment tool (Table S8)?

**Authors’ response:** Descriptive studies, which do not explore the relationship between ecological factors and TB incidence or mortality, were not included in this analysis. Our goal was to examine how ecological factors are associated with TB incidence and mortality, so only studies that addressed these associations were considered. Consequently, descriptive studies and other full-text articles that did not meet our inclusion criteria were not assessed for quality. We applied the quality assessment tool outlined in Table S8 exclusively to the studies that were included, to ensure the validity of our results.

**Comment 3:** “Quality Assessment”, p.5: Please put this section after “Statistical analysis” to be in line with how the results are presented. Lines 172-174 on Egger’s regression test on publication bias can also be added to this section.

**Authors’ response:** The order is now reorganized as suggested. **Please see lines 171-178.**

**Comment 4:** Lines 168-174: It would be good to add a line or two to support the use of random effects meta-analysis here. You can reference this paper:Riley RD, Higgins JP, Deeks JJ. Interpretation of random effects meta-analyses. BMJ2011;342:d549. doi:10.1136/bmj.d549. pmid:21310794**.** It mentions that “heterogeneity in treatment effects is caused by differences in study populations (such as age of patients), interventions received (such as dose of drug), follow-up length, and other factors”. In your analysis, the different spatial scales (e.g. county-level, provincial-level) of the studies could be an important factor influencing the treatment effects. Please also note that the pooled result from a random-effects meta-analysis should be interpreted as the average of the intervention effects across studies and not an estimate of the common effect.

**Authors’ response:** We have included additional sentences as follows “…. Random effects meta-analysis is particularly valuable when synthesizing results from studies with varying underlying populations or methodologies. Random effects meta-analysis accounts for both within-study and between-study variability, and enhances the robustness of the conclusions drawn……” **please refer to lines 164-167.**

**Comment 5:** Line 175, p.6: “Results” can be a new section, containing the subsections “Characteristics of included studies”, “Ecological level factors associated with TB incidence”, “Ecological factors associated with TB mortality”. So, the main sections in the paper would be “Introduction”, “Methods”, “Results” and “Discussion”.

**Authors’ response:** We have now assigned **“**Results” as main section and "Characteristics of included studies", "Ecological level factors associated with TB incidence", and "Ecological factors associated with TB mortality" as subsections. **Kindly refer to the result section on pages 6,7, 12, and 15.**

**Comment 6:** Line 178, p.6: It seems that the total number of references here is 52 instead of 53.

**Authors’ response:** This was because one publication (Sohn 2019) has compiled two studies or two outcomes. This article investigated and reported factors associated with TB incidence and mortality and we considered this study per outcome. However, to avoid this confusion we have now mentioned this information in the result and reported the overall total number of studies included as 52 studies.

**Comment 7:** Table 1: Please be consistent with the terms used. E.g. use “Negative” instead of “Protective”, and “Positive” instead of “Risk”. Could you also indicate which of these rows were used for the meta-analysis?

**Authors’ response:** We have now ensured consistent use of the terms “positive” and “negative” throughout the document. Additionally, we have indicated the studies included in the meta-analysis with an asterisk in Table 1. Please note that the number of studies included in the meta-analysis has been mistakenly written as 26 and we have now corrected it to 24. Sorry for the confusion created by this typological error. **Kindly refer to the footnote in Table 1.**

**Comment 8**: p.12, “Ecological level factors associated with TB incidence”: It may be better to present the narrative synthesis results before the meta-analysis results, to be in line with the flow of the "Methods" section. You can also draw links between the two results to provide credibility to your meta-analysis results. E.g. The pooled adjusted relative risk for average relative humidity is greater than 1 which is in line with the mainly positive association seen from the narrative synthesis.

**Authors’ response:** We have now presented narrative synthesis results before meta-analysis results as suggested. To be in line with subsequent sections and to further improve the flow of the manuscript we have presented narrative synthesis results before the meta-analysis results in the abstract as well. **Please refer to the result section lines 267 and abstract.**

**Comment 9:** Line 240, p.13: The phrase “increased the occurrence of TB” seems to imply causality; however, I think only association was established. Please rephrase this.

**Authors’ response:** We have now replaced the term “occurrence” with “incidence” and rephrased as “…was associated with increased incidence of TB….” **Please refer to line 223.**

**Comment 10:** Lines 290-296, p.16: Will you be sharing the individual item scores for each paper (e.g. in a supplementary Excel file)? This will enable the readers can better understand the common biases mentioned.

**Authors’ response:** We have now provided the individual item scores across three domains for each paper in a Microsoft Excel file as suggested. **Please see citation in line 296.**

**Comment 11:** P.16-17, “Discussion”: The second paragraph on relative humidity and rainfall is confusing. Based on the narrative synthesis, high average relative humidity and high average rainfall are consistently associated with increased TB incidence. This is in line with the meta-analysis results. However, in lines 324-325, you mention that “Low rainfall is also associated with an increased risk of TB incidence”. Are you mentioning an exception to the rule? Is this context/location-dependent, and possibly related to the publication bias you mentioned on line 300?

**Authors’ response**: Thank you for your observation. In our narrative synthesis, increased rainfall correlates with a higher incidence of TB, aligning with the meta-analysis results. We have now clarified this in the discussed. Please **kindly refer to lines 327-331 of discussion**.

**Comment 12:** Lines 387-388: Please provide a reference for this respiratory disease study.

**Authors’ response:** We have now provided the citation. **Please see line 390.**

**Comment 13**: Lines 388-390: Could you provide examples of the kinds of subgroup analyses you would consider if you had more studies? E.g. identifying similar characteristics in the locations associated with positive/negative effects and identifying the combinations of covariates used in the models (i.e. exploring potentially confounding factors) may be useful.

**Authors’ response:** Mainly conducting subgroup analysis by region (i.e, Africa e.t.c) or country (high TB burden) was important to show how TB risk factors vary by location. We have now included this idea as suggested. **Kindly refer to the limitation section in lines 392-396.**

**Comment 14:** There were several instances where “Poisson” was misspelt: on line 141 of p.5, line 194 of p.7 in Table S8 and multiple times in Table 1. Please correct these.

**Authors’ response:** We have now corrected this in the revised manusicpt.

**Reviewer’ comments**

1. If the authors have adequately addressed your comments raised in a previous round of review and you feel that this manuscript is now acceptable for publication, you may indicate that here to bypass the “Comments to the Author” section, enter your conflict-of-interest statement in the “Confidential to Editor” section, and submit your "Accept" recommendation.

**Reviewer #2**: All comments have been addressed.

2. Does this manuscript meet PLOS Global Public Health’s [publication criteria](https://journals.plos.org/globalpublichealth/s/criteria-for-publication)? Is the manuscript technically sound, and do the data support the conclusions? The manuscript must describe methodologically and ethically rigorous research with conclusions that are appropriately drawn based on the data presented.

**Reviewer #2**: Yes

3. Has the statistical analysis been performed appropriately and rigorously?

**Reviewer #2**: Yes

4. Have the authors made all data underlying the findings in their manuscript fully available (please refer to the Data Availability Statement at the start of the manuscript PDF file)?
The [PLOS Data policy](https://journals.plos.org/globalpublichealth/s/data-availability) requires authors to make all data underlying the findings described in their manuscript fully available without restriction, with rare exception. The data should be provided as part of the manuscript or its supporting information or deposited to a public repository. For example, in addition to summary statistics, the data points behind means, medians and variance measures should be available. If there are restrictions on publicly sharing data—e.g. participant privacy or use of data from a third party—those must be specified.

**Reviewer #2**: Yes

5. Is the manuscript presented in an intelligible fashion and written in standard English?
PLOS Global Public Health does not copyedit accepted manuscripts, so the language in submitted articles must be clear, correct, and unambiguous. Any typographical or grammatical errors should be corrected at revision, so please note any specific errors here.

**Reviewer #2**: Yes

6. Review Comments to the Author

Please use the space provided to explain your answers to the questions above. You may also include additional comments for the author, including concerns about dual publication, research ethics, or publication ethics. (Please upload your review as an attachment if it exceeds 20,000 characters)

**Reviewer #2**: Comments have been addressed.

**Authors’ response:** Thank you.
